# Overview: an iPS cell stock at CiRA

**DOI:** 10.1186/s41232-019-0106-0

**Published:** 2019-09-02

**Authors:** Masafumi Umekage, Yoshiko Sato, Naoko Takasu

**Affiliations:** Department of Fundamental Cell Technology, Center of iPS Cell Research and Application (CiRA), Kyoto University, 53 Kawahara-cho, Shogoin, Sakyo-ku, Kyoto, 606-8507 Japan

**Keywords:** iPS cells, Stock, Clinical-grade, HLA, Regenerative medicine

## Abstract

Induced pluripotent stem cells (iPSCs) can be produced from various somatic cells and have the ability to differentiate into various cells and tissues of the body. Regenerative medicine using iPSCs is expected to manage diseases lacking effective treatments at present. We are establishing a safe and effective iPSC stock that can be used for regenerative medicine. Our iPSC stock is recruited from healthy, consenting HLA-type homozygous donors and is made with peripheral blood-derived mononuclear cells or umbilical cord blood. We hope to minimize the influence of immune rejection by preparing HLA homozygous iPSCs. Our stock is made at the Cell Processing Center (CPC), Center for iPS Cell Research and Application (CiRA). We are preparing iPS cells that maximize matching of the Japanese population at the major HLA loci. This iPSC stock is intended to be offered not only to Japanese centers but also overseas medical institutions and companies. In August 2015, we began offering the iPSC stock for regenerative medicine and now offer 21 clones made from 5 donors.

## Background

### Overview of the iPSC stock project

iPSCs have the ability to self-renew and differentiate into all cell types of the human body, and their practical application is expected to be realized in the near future [1, 2]. There are two approaches to iPSC-based cell transplantations: autologous and allogeneic. Autologous transplantation is a procedure in which target cells/tissues derived from an individual’s own iPSCs are transplanted into the self. In contrast, allogeneic transplantation uses iPSC-derived target cells/tissues donated by a third person for the transplantation.

Autologous transplantation is preferable in order to avoid immune reactions and unknown virus infections. However, it is extremely costly, and the time required to prepare and transplant the target cells/tissues can be unacceptably long medically. In September 2014, for the world’s first iPSC-based therapy, the autologous transplantation of iPSC-derived retinal pigment epithelium (RPE) cells required the patient to wait over 10 months for the surgery and cost nearly 100 million yen (approximately US $1 million) [3]. With an allogeneic approach, a sufficient number of iPSCs can be generated and comprehensive quality testing can be performed in advance, which can help reduce the cost and time of the iPSC manufacturing process.

However, immune rejection remains an issue in allogeneic transplantation. HLA antigens have been known to play an important role in immune responses. In hematopoietic stem cell transplantation (HSCT), HLA matching between donors and recipients lowers the risk of graft rejection and graft-versus-host disease (GVHD). Moreover, for some organ transplantations, HLA matching has been shown to enhance allograft survival in adults who receive an organ from either a deceased or living donor. Following these models, we are screening healthy donors with homozygous human leukocyte antigen (HLA)-A, HLA-B, and HLA-DR to establish our iPSC stock. Based on previous works [4, 5], it is estimated that 10, 75, and 140 cell lines would match approximately 50%, 80%, and 90% of the Japanese population, respectively.

We have released clinical-grade iPSCs from the stock, including three lines from peripheral blood mononuclear cells (PBMCs) with first-, second-, and third-ranked HLA haplotypes and two lines from cord blood with first-ranked HLA haplotypes at the Facility for iPS Cell Therapy (FiT), a cell-processing center (CPC) at the Center for iPS Cell Research and Application (CiRA), Kyoto University, Japan. These three haplotypes cover approximately 32% of the Japanese population. One study using cells from our iPSC stock showed the effectiveness of this strategy in non-human primates [6]. Accordingly, cells from the stock were used in the first iPSC-related allogeneic transplantation, which also used RPE cells and followed the same procedure as the above autologous transplantation. The surgery time was shortened to about 1 month, and the overall cost was under 20 million yen per patient [7]. However, despite these encouraging results, the overall utility of regenerative medicine with HLA matching needs more investigation [8–10].

## Donor recruitment

Donor eligibility for the iPSC stock is conditional on 3 HLA loci (HLA-A, HLA-B, and HLA-DR) being homozygous. To achieve our goals, hundreds of thousands of people would need to be tested for HLA typing if randomly selected from the population to identify dozens of HLA homozygous donors. Therefore, we are in collaboration with the Japan Red Cross, Japan Marrow Donor Program, and several Japanese cord blood banks because they have already performed HLA typing for huge numbers of people. In this collaboration, the partner institutes deliver an overview of our iPSC stock project to the HLA homozygous candidates, and the candidates decide whether or not to take part in our project. As a result, 36 donors (24 haplotypes) have agreed to donate blood to our project. In addition to the homozygosity described above, our donors are homozygous for 2 or 3 of HLA-C, HLA-DQ, and HLA-DP as well. Overall, of the 36 donors, 20 donors were homozygous for all 6 HLA loci, and 15 donors were homozygous for the 5 HLA loci (Table 1).

**Table 1:** Status of donor recruitment

Ranking of haplotype frequency	Haplotypes (HLA-A, HLA-B, HLA-DR)	Number of matching HLA homo(max. 6)
Peripheral blood		
1	A*24:02-B*52:01-C*15:02	6
1	A*24:02-B*52:01-C*15:02	5
1	A*24:02-B*52:01-C*15:02	5
1	A*24:02-B*52:01-C*15:02	6
1	A*24:02-B*52:01-C*15:02	6
1	A*24:02-B*52:01-C*15:02	6
2	A*33:03-B*44:03-C*13:02	6
3	A*24:02-B*07:02-C*01:01	6
4	A*24:02-B*54:01-C*04:05	6
5	A*02:07-B*46:01-C*08:03	5
6	A*11:01-B*15:01-C*04:06	5
7	A*24:02-B*59:01-C*04:05	6
8	A*11:01-B*54:01-C*04:05	6
9	A*24:02-B*40:06-C*09:01	5
10	A*26:01-B*40:02-C*09:01	5
11	A*24:02-B*51:01-C*09:01	6
13	A*26:02-B*40:06-C*09:01	5
14	A*24:02-B*46:01-C*08:03	5
17	A*02:06-B*35:01-C*15:01	5
18	A*24:02-B*40:02-C*09:01	5
21	A*24:02-B*40:01-C*09:01	6
22	A*02:06-B*39:01-C*15:01	6
23	A*26:01-B*40:02-C*08:02	3
24	A*24:02-B*40:01-C*04:05	6
26	A*02:07-B*46:01-C*09:01	5
43	A*26:02-B*15:01-C*14:06	6
51	A*02:01-B*40:02-C*09:01	6
66	A*02:01-B*46:01-C*08:03	6
98	A*11:01-B*15:01-C*09:01	5
Umbilical cord blood		
1	A*24:02-B*52:01-C*15:02	6
1	A*24:02-B*52:01-C*15:02	6
1	A*24:02-B*52:01-C*15:02	6
1	A*24:02-B*52:01-C*15:02	5
2	A*33:03-B*44:03-C*13:02	6
2	A*33:03-B*44:03-C*13:02	5
2	A*33:03-B*44:03-C*13:02	5

Thirty-six donors (24 haplotypes) agreed to be involved in our project. Five donor-derived iPSC stocks have already been released. All of our homozygous donors with 3 HLA loci are also homozygous for 2 or 3 other HLA loci: HLA-C, HLA-DQ, or HLA-DP

## Manufacturing

We collect blood at three major cities in Japan (Kyoto, Nagoya, and Tokyo) from HLA homozygous donors who have given their informed consent to participate in our iPSC stock project. PBMCs are isolated from the donor peripheral blood and cryopreserved at FiT on the same day of the blood collection.

**Fig. 1:**
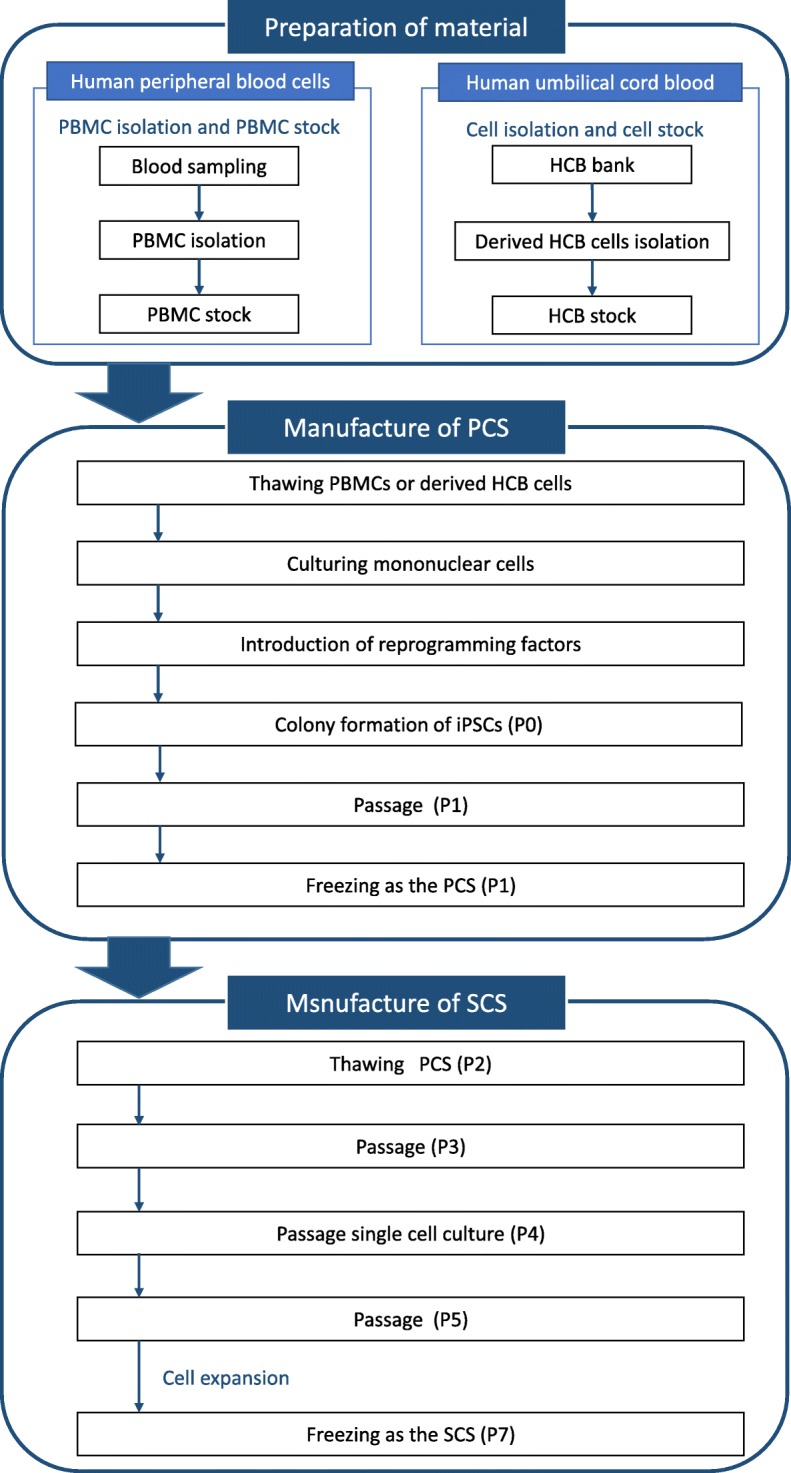
Production flow of an iPS cell stock. Production is largely composed of the preparation of the materials (human peripheral blood mononuclear cells (PBMCs) and human umbilical cord blood (HCB)), the PCS and the SCS. The PCS is made from cultured cells, which are then gene-transferred to colony formation and stored at P1. For the SCS, the PCS is recultured and then frozen at P7

In accordance with the predetermined production schedule, the cryopreserved PBMCs or cord blood are thawed, cultured, and gene-transferred with hOCT3/4, mp53DD, hSK, hUL, and EBNA1 by the electroporation method [4, 11–14]. After expansion culture using StemFiT AK03 medium and the iMatrix-511 system, several iPSC colonies are confirmed in approximately 25 days [15, 16]. All of the colonies are detached, suspended, dispensed into several tubes as a primary cell stock (PCS), and cryopreserved (Fig. 1).

After re-culture of the PCS, 1 or 10 cells are plated into a single well of a plate in order to select cell lines with fewer remaining plasmids and subcultured. Twelve to 15 cell lines as appropriate are selected and dispensed into approximately 70 tubes as a secondary cell stock (SCS) (Fig. 1). Originally, we were unable to predetermine the total number of passages necessary for SCS production, because residual plasmids were reduced by passages in cell culture. Our current method is expected to improve the plasmid clearance and shorten the manufacturing period.

We also manufacture a research-grade iPSC stock, which is branched in the middle of the SCS manufacturing and expanded in a non-CPC environment. We ask users to use the research-grade iPSC stock initially, because the clinical-grade stock is limited.

## Quality testing

We conduct two types of clinical testing: release assays and reference assays. Release assays are defined as mandatory testing for shipping and include contamination tests, such as sterility and viral testing, morphology, and HLA and STR analyses to prevent mix-ups. Reference assays are important tests in which the criteria cannot be standardized but the result affects the product release if any abnormalities are identified (Table 2). These tests include the evaluation of vector clearance, genomic mutations, specific markers for undifferentiated cells, and post-thaw proliferative capacity [17, 18]. A genomic analysis is crucial, because it would confirm genomic mutations in not only iPSCs but also the differentiated cells. We perform a comprehensive genomic analysis on our SCS using whole-exome sequencing for SNV/Indel detection, an SNP array for copy number variations (CNV) detection and whole-genome sequencing for SNV/Indel and CNV detection [19–26]. Based on the results of the reference and release assays, the clinical-grade stock is selected.

**Table 2:** Quality tested items

Status	Test item	Method	Criteria
Release assay	Morphology	Microscopy	ES cell-like
	HLA	PCR-SBT	Match with origin
	STR	PCR and capillary electrophoresis	Match with origin
	Bacteriology	BacT/ALERT	Negative
	Viral testing	PCR	Negative
	Endotoxin	Kinetic turbidimetric	≦ 5 EU/mL
	Mycoplasma	PCR	Negative
Reference assay	Pluripotent marker expression	Flow cytometry	–
		Microarray	–
	SNV/Indel	WGS/WES	–
	CNV	WGS/SNP array	–
	Analysis of genetic variation	WGS	
	Karyotype	Conventional Giemsa staining	–
		G-banding	–
	Residual vector	PCR (Taqman qPCR)	–
	Cell number and viability immediately after thawing	Cell count	–
	Average of doubling time among 3 passages of subculture	Cell count	–
	Cell number and viability after 6–7 days culture of thawed cells	Cell count	–

A release assay is defined as mandatory testing that must be carried out before shipping. A reference assay is defined as an important test for which the criteria cannot be standardized, but the results may delay the product release if any abnormality is identified

Because of the manufacturing capability, no more than six cell lines are selected. Therefore, some lines without abnormalities are still not released. The selected lines undergo an overall review for release based on the results of the release testing and manufacturing process. In addition, we perform a whole-genome analysis, methylation analysis, and single-cell analysis on the iPSCs immediately before differentiation, along with testing the differentiated cells for tumorigenicity and the final product for safety in animal models before conducting clinical research and clinical trials under the collaboration research agreement with the partner institutes. In this way, we can obtain comprehensive data for iPSCs and all kinds of differentiated cells. These findings will help improve the safety of iPSC-related products.

## Project achievements

In August 2015, CiRA released the first clinical-grade iPSC stock. It was the QHJI cell line, which had the most frequent HLA haplotype in Japan. As mentioned above, five donor-derived iPSC stocks have been released, which cover approximately 32% of the Japanese population. We have provided clinical-grade iPSC stocks for 12 projects performed by academia and companies and research-grade iPSC stocks for a further 32 projects. In March 2017, as mentioned above, the first-in-human allogeneic transplantation using our QHJI line (RPE cells) was performed at Kobe City Medical Center General Hospital in collaboration with Osaka University, RIKEN, and CiRA.

## Issues to be resolved

Our partner institutes have their own differentiation protocols for selecting the best of several candidate lines. As a result, differences in the differentiation efficacy and potency among the same donor and similar cell lines have come to light. These differences in differentiation capability might be due to differences among donors, cell lines, or culture techniques among institutes, but the details remain unclear [27, 28]. This problem must be resolved for further progress in iPSC-based regenerative medicine.

## Conclusions

Our iPSC stock has the potential to reduce immune reactions to a minimum. At present, we have established an iPSC stock from PBMCs or cord blood of healthy HLA homozygous donors. Our iPSC stock can cover approximately 32% of the Japanese population currently, but the percentage is expected to increase with time. We will provide our iPSC stock to not only domestic institutes but also overseas institutes to support the clinical application of iPSC-based therapy.

## Data Availability

Not applicable

## Abbreviations

CiRA: Center for iPS Cell Research and Application
CNV: Copy number variations
CPC: Cell processing center
FiT: Facility for iPS Cell Therapy
GVHD: Graft rejection and graft-versus-host disease
HCB: Human umbilical cord blood
HLA: Human leukocyte antigen
HSCT: Hematopoietic stem cell transplantation
iPSCs: Induced pluripotent stem cells
PBMC: Peripheral blood mononuclear cell
PCR-SBT: PCR-sequence-based typing
PCS: Primary cell stock
RPE: Retinal pigment epithelium
SCS: Secondary cell stock
SNV/Indel: Single nucleotide variant/insertion and deletion
STR: Short tandem repeat
WGS: Whole genome sequencing
WES: Whole exome sequencing

## References

[1] Takahashi K (2007). Induction of pluripotent stem cells from adult human fibroblasts by defined factors. Cell..

[2] Takahashi K (2006). Induction of pluripotent stem cells from mouse embryonic and adult fibroblast cultures by defined factors. Cell..

[3] Mandai M (2017). Autologous induced stem-cell–derived retinal cells for macular degeneration. N Engl J Med..

[4] Okita K (2011). A more efficient method to generate integration-free human iPS cells. Nature Methods..

[5] HLA Laboratory. http://hla.or.jp/med/frequency_search/en/haplo/ [accessed 30th November 2018]

[6] Morizane A (2017). MHC matching improves engraftment of iPSC-derived neurons in non-human primates. Nat Commun..

[7] The Japan Times. 2017. Japanese team conducts world’s first transplant of iPS cells. Accessed 29 Mar 2017.

[8] Ichise H (2017). NK cell alloreactivity against KIR-ligand-mismatched HLA-haploidentical tissue derived from HLA haplotype-homozygous iPSCs. Stem Cell Reports..

[9] Zhao T (2011). Immunogenicity of induced pluripotent stem cells. Nature..

[10] Okita K (2011). Immunogenicity of induced pluripotent stem cells. Circ Res..

[11] Okita K (2010). Generation of mouse-induced pluripotent stem cells with plasmid vectors. Nat Protoc..

[12] Nakagawa M (2010). Promotion of direct reprogramming by transformation-deficient Myc. PNAS..

[13] Siemen H (2005). Nucleofection of human embryonic stem cells. Stem Cells Dev..

[14] Yu J (2009). Human induced pluripotent stem cells free of vector and transgene sequences. Science..

[15] Nakagawa M (2014). A novel efficient feeder-free culture system for the derivation of human induced pluripotent stem cells. Sci Rep..

[16] Miyazaki T (2012). Laminin E8 fragments support efficient adhesion and expansion of dissociated human pluripotent stem cells. Nat Commun..

[17] International Stem Cell Initiative, et al. (2007). Characterization of human embryonic stem cell lines by the International Stem Cell Initiative. Nat Biotechnol..

[18] Koyanagi-Aoi M (2013). Differentiation-defective phenotypes revealed by large-scale analyses of human pluripotent stem cells. Proc Natl Acad Sci U S A..

[19] Yoshida K (2011). Frequent pathway mutations of splicing machinery in myelodysplasia. Nature..

[20] Shiraishi Y (2013). An empirical Bayesian framework for somatic mutation detection from cancer genome sequencing data. Nucleic Acids Res..

[21] Rausch T (2012). DELLY: structural variant discovery by integrated paired-end and split-read analysis. Bioinformatics..

[22] Koboldt DC (2012). VarScan 2: somatic mutation and copy number alteration discovery in cancer by exome sequencing. Genome Res..

[23] Wang K (2007). PennCNV: an integrated hidden Markov model designed for high-resolution copy number variation detection in whole-genome SNP genotyping data. Genome Res..

[24] Gogarten SM (2012). GWASTools: an R/Bioconductor package for quality control and analysis of genome-wide association studies. Bioinformatics..

[25] González JR (2011). A fast and accurate method to detect allelic genomic imbalances underlying mosaic rearrangements using SNP array data. BMC Bioinformatics..

[26] Pique-Regi R (2010). R-Gada a fast and flexible pipeline for copy number analysis in association studies. BMC Bioinformatics..

[27] Bock C (2011). Reference Maps of human ES and iPS cell variation enable high-throughput characterization of pluripotent cell lines. Cell..

[28] Newman AM (2010). Lab-specific gene expression signatures in pluripotent stem cells. Cell Stem Cell..

